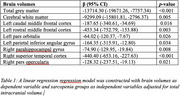# The Cognitive and Imaging Correlates of Sarcopenia: Insights from an Urban Indian Cohort

**DOI:** 10.1002/alz70860_106818

**Published:** 2025-12-23

**Authors:** Thomas Gregor Issac, Monisha S, Latha Diwakar

**Affiliations:** ^1^ Centre for Brain Research (CBR), Bangalore‐12, Bangalore, Karnataka, India; ^2^ Centre for Brain Research, Indian Institute of Science, Bengaluru, Karnataka, India

## Abstract

**Background:**

Sarcopenia is associated with an increased risk of cognitive impairment, yet very few studies have examined structural brain changes in sarcopenia. The current study aims to explore cognitive changes and their neuroimaging correlates of individuals with sarcopenia in an urban Indian cohort.

**Method:**

Cognitively healthy participants (*N* = 1119) from the Tata Longitudinal Study of Aging were screened for sarcopenia using two parameters: calf circumference (M < 34cm, F < 33cm) and handgrip strength (M < 28kg, F < 18kg). Based on these, participants were classified into two groups namely, healthy (satisfying neither criterion), and sarcopenia (satisfying both criteria). Cognitive function was assessed using Addenbrooke's Cognitive Examination‐III (ACE‐III). A 3T magnetic resonance imaging equipment was used for brain imaging. Generalized linear model was used to find the association between cognition, brain volumes, and sarcopenia.

**Result:**

The generalized linear model revealed that the sarcopenia group performed poorer in tests of global cognition, attention, and language domains. Neuroimaging results revealed that overall grey and white matter volumes were lesser in sarcopenia group. Furthermore, we found that the volumes of brain regions associated with attention and language functions were lesser in the sarcopenia group when compared to healthy controls (Table 1).

**Conclusion:**

Preventing sarcopenia through lifestyle modifications, risk‐factor control, and timely interventions, play a significant role in preventing brain volume loss and cognitive decline with aging.